# A non-small cell lung cancer fragile elderly patient treated with immunotherapy and non-ablative radiation therapy: a case report of a winning combination

**DOI:** 10.3389/fonc.2025.1642564

**Published:** 2025-08-15

**Authors:** Jessica Saddi, David Alberto Santos Hernandez, Giulia Maria Stella, Giulia Galli, Sabrina Borgetto, Elisabetta Bonzano, Andrea Lancia, Salvatore La Mattina, Sara Colombo, Luigi Squillace, Guido Baietto, Chandra Bortolotto, Gioacchino D’Ambrosio, Laura Mantovani, Paolo Pedrazzoli, Francesco Agustoni

**Affiliations:** ^1^ San Matteo Hospital Foundation (IRCCS), Pavia, Italy; ^2^ Department of Radiation Oncology, Centro Nacional de Radioterapia, San Salvador, El Salvador; ^3^ Department of Radiation Oncology, Fondazione IRCCS Policlinico San Matteo, Pavia, Italy; ^4^ Department of Medical Research, Instituo Nacional de Salud, San Salvador, El Salvador; ^5^ Unit of Respiratory Diseases, Cardiothoracic and Vascular Department, IRCCS Policlinico San Matteo, Pavia, Italy; ^6^ Department of Internal Medicine and Medical Therapeutics, University of Pavia Medical School, Pavia, Italy; ^7^ Department of Medical Oncology, Fondazione IRCCS Policlinico San Matteo, Pavia, Italy; ^8^ Unit of Thoracic Surgery, Cardiothoracic and Vascular Department, IRCCS Policlinico San Matteo, Pavia, Italy; ^9^ Diagnostic Imaging and Radiotherapy Unit, Department of Clinical, Surgical, Diagnostic and Pediatric Sciences, University of Pavia, Pavia, Italy; ^10^ Radiology Institute, Fondazione IRCCS Policlinico San Matteo, Pavia, Italy; ^11^ Pathology Unit, Department of Diagnostical Services and Imaging, Fondazione IRCCS Policlinico San Matteo, Pavia, Italy; ^12^ Department of Medical Physics, Fondazione IRCCS Policlinico San Matteo, Pavia, Italy

**Keywords:** NSCLC, immunotherapy, radiotherapy, non-ablative SBRT, SBRT

## Abstract

**Background:**

Radiation therapy is used in the clinical scenario of oligo-metastatic lung cancer as a weapon to delay the subsequent line of systemic therapy, particularly in the case of oligo-progressive disease. In this setting, the integration of immunotherapy and radiotherapy plays an important role to achieve local control and improve progression-free survival (PFS).

**Case presentation:**

We reported the case of an elderly fragile patient affected by advanced non-small cell lung cancer treated with pembrolizumab as first systemic line and immuno-modulant radiation therapy at oligo-progression. More specifically, he underwent stereotactic body radiation therapy using non-ablative regimen (24 Gy in 3 fractions) achieving partial response with abscopal effect and without drug interruption. After one year, during immunotherapy mediastinal and parenchymal progression occurred and he received another radiation treatment using conventional non-ablative regimen (40 Gy in 20 fractions). Complete response was observed without severe side effects (his poor respiratory function did not change during both treatments).

**Conclusion:**

In this case report we showed that the association of immunotherapy and non-ablative radiation regimens may represent a safe and effective strategy to achieve complete response also in fragile patients, in whom the burden of side effects should be prioritized.

## Introduction

Immunomodulatory radiotherapy (iRT) refers to the use of ionizing radiation with the goal of positively modulating immune system activation, promoting the recognition of tumor cells through a mechanism similar of that usually triggered by vaccines (1–3), inducing a loco-regional and systemic immune-mediated response (abscopal effect) and consequently achieving a synergistic effect when combining with immunotherapeutic agents.

Patients with metastatic non-oncogene addicted non-small cell lung cancer (NSCLC) are generally treated with systemic therapy (immune check-point inhibitors alone or in combination with chemotherapy according to anti programmed death-ligand 1 status -anti-PD-L1-) as first-line treatment approach (4).Pembrolizumab (anti-PD-L1) received approval in 2016 in the first line setting as a single agent for patients whose tumors have high PD-L1 expression (tumor proportion score of >50%). The efficacy of pembrolizumab in combination with platinum-based chemotherapy was also demonstrated in several large phase III randomized trials in patients with metastatic NSCLC regardless of PD-L1 expression level (5, 6). As reported in registrative clinical trials, the median PFS of pembrolizumab as monotherapy in PD-L1 overexpressed patients is 10.3 months (7–9) and 8.8 months (5, 10) when used in combination with chemotherapy for non-squamous NSCLC.

In cases of oligo-progression, radiotherapy (RT) is often added to systemic therapy and plays an important role in disease control. In clinical practice RT in terms of hypo-fractionated regimen or stereotactic ablative therapy (SABR) is common used and well tolerated with concomitant immunotherapy (IO).

Recently, a systematic review focused on reporting the distant radiobiological effects (abscopal or bystander effect) of stereotactic body radiation therapy (SBRT) (11); most of these responses are reported for melanoma (24%) and NSCLC (13%) patients and occurred more frequently when using sub-ablative hypo-fractionated doses and concomitant IO.

At the moment, there are no clear recommendations on the use of sub-ablative doses in a radical treatment setting for patients with oligo-progression undergoing IO and SABR is generally used in this setting. However, in patients with pulmonary comorbidities such as severe obstructive pulmonary disease (COPD) or interstitial lung diseases, where treatment-related radiotoxicity may be a significant concern, sub-ablative dosing may represent an opportunity for systemic disease control.

This case report presents a patient affected by advanced NSCLC and concomitant severe COPD/emphysema who started pembrolizumab as first-line systemic therapy and then developed oligoprogression at two different time points. In both instances, iRT treatment with sub-ablative doses was administered, achieving complete response (CR) and continuation of the same therapy for up to 45 months.

## Case report

**Figure 1:**
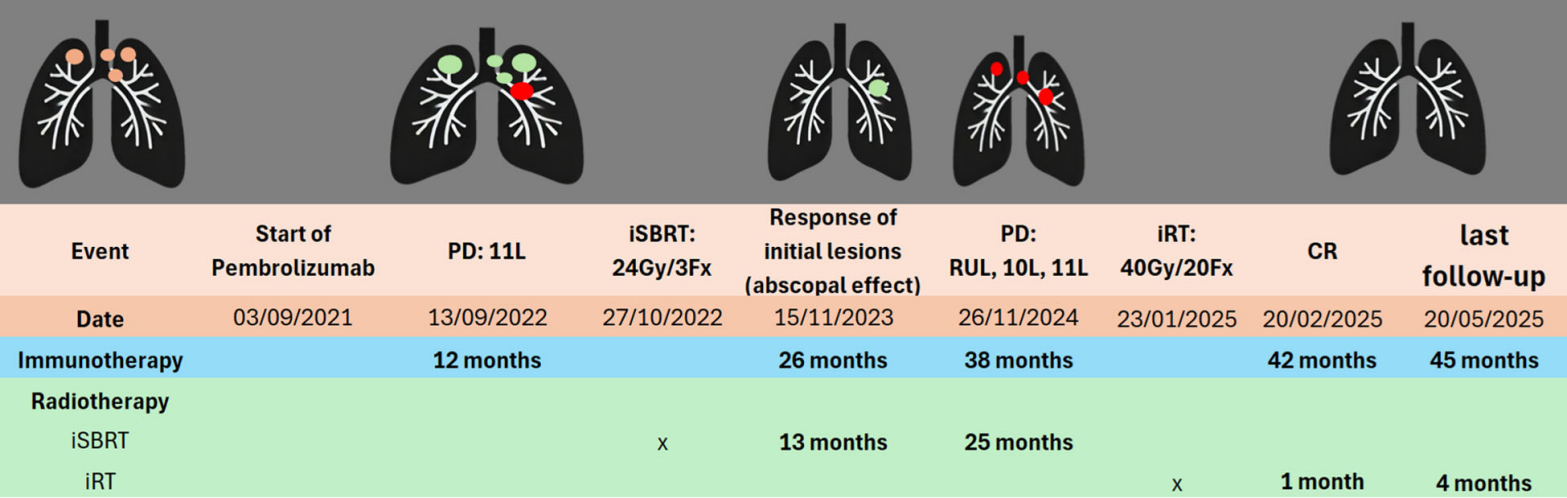
Timeline of treatments. Initial lesions 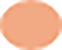 SD 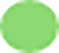 PD 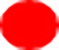. SD, Stable disease; PD, Progressive disease; CR, Complete response; iSBRT, immune-modulating stereotactic body radiotherapy; iRT, immune-modulating radiotherapy; 11L, Left lymph node station 11; 10L, Left lymph node station 10; RUL, Right upper lobe.

In 2021 a former smoker (40 pack-year) 76-year-old male patient, with a performance status of 1, according to eastern cooperative oncology group (ECOG), with very severe COPD (GOLD 4), emphysema (Goddard 18 points) and cardio-vascular comorbidities (arterial hypertension, history of transient ischemic attack and paroxysmal atrial fibrillation in treatment with oral anticoagulant), received diagnosis of bilateral lung adenocarcinoma in the upper lobes and positive mediastinal lymph nodes (cT1b cN2 M1a; stage IV according to AJCC VIII edition, **Figure 1**) based on tomography scan (CT scan); positron emission tomography (PET) and endobronchial ultrasound-guided transbronchial needle aspiration (EBUS-TBNA). The immunohistochemical analysis revealed a PD-L1 tumor proportion score (TPS) of more than 50%, and the genetic analysis with next-generation sequencing (NGS) of *EGFR, KRAS, BRAF, LKB1, ERBB2* and *MET* did not reveal any targetable mutations.

Following multidisciplinary discussion, the patient was considered as oligometastatic setting but not suitable for radical local treatment due to the mediastinal lymph nodes involvement. Thus he started pembrolizumab, as first-line systemic treatment, 200 mg each three weeks (q3w), reporting partial response which was maintained for one year.

After 12 months, the patient experienced oligo-progression at lymph node station 11L, confirmed trough computed tomography (CT). Considering the pulmonary status and the ongoing IO treatment, an immune-modulating stereotactic body radiotherapy (iSBRT) treatment was planned to target only the site of progression, with a sub-ablative dose of 24 Gy in three fractions to the 80% isodose to the planning target volume (PTV), using volumetric modulated arc therapy (VMAT) without drug interruption. Prior to treatment, the patient underwent pulmonary spirometry showing permissive results for treatment as shown below (**Table 1**).

**Table 1:** Patient spirometric data by year of evaluation.

Year	*Oct 2022	May 2023	*Apr 2024	Apr 2025
FEV1; L	0.098	2.02	2	1.97
DLCO; % of predicted value	40%	44%	51%	40%

FEV1, forced expiratory volume in 1 second. DLCO: diffusing capacity of the lung for carbon monoxide.

*prior to radiotherapy.

A CR was documented 13 months from iSBRT in the untreated lesions, while the irradiated lymph node station was stable; this response was maintained for other 12 months.

In November 2024, 25 months after iSBRT, mediastinal and lung oligo-progression was detected at stations 10L, 11L (pre-treated site) and right upper lobe (RUL), confirmed by PET-CT.

**Figure 2:**
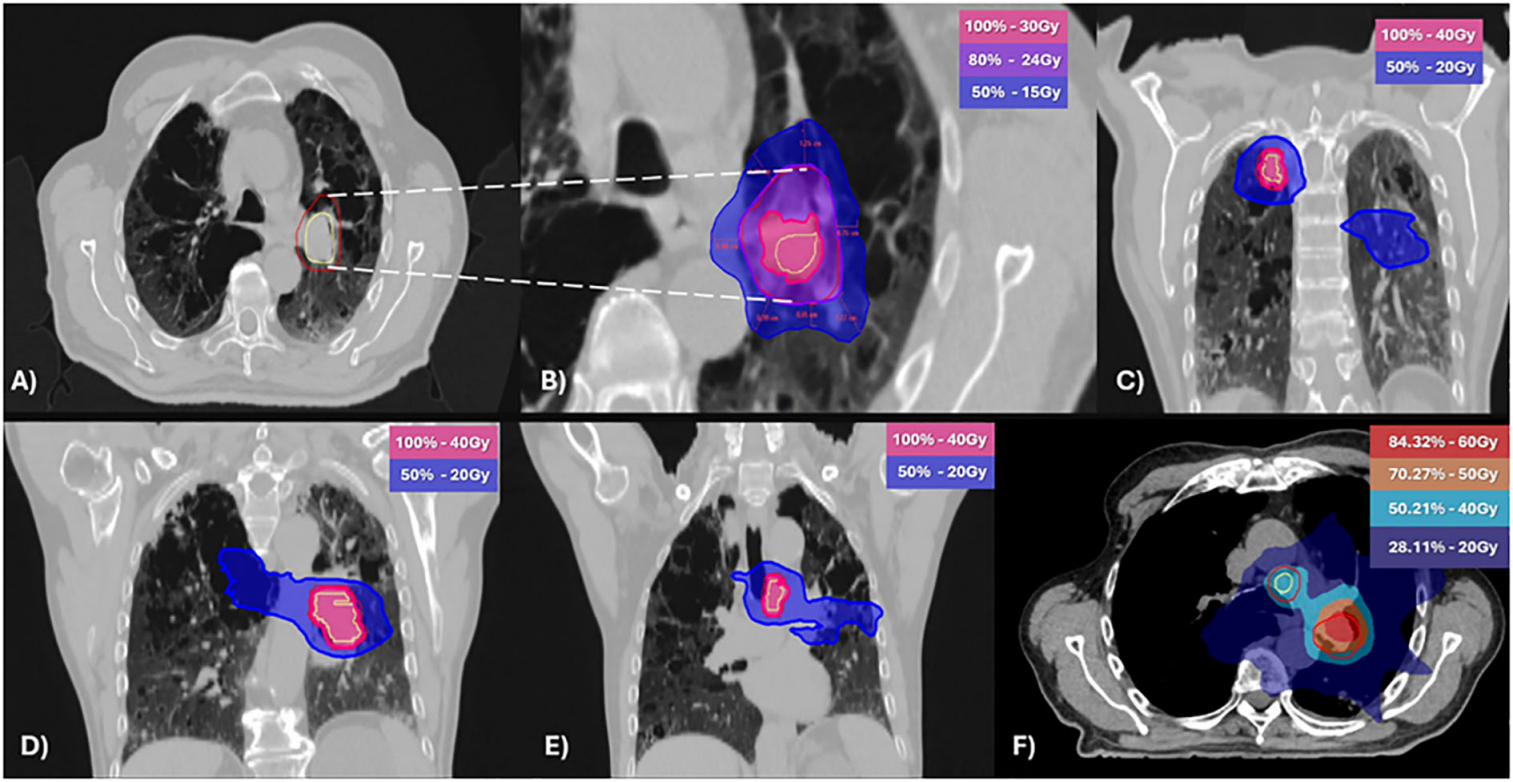
Stereotactic body radiotherapy [SBRT; **(A, B)**] and normo-fractionated radiotherapy **(C-E)** plans with geometrical dose accumulation **(F)**. **(A)** Clinical target volume (CTV) in yellow and planning target volume (PTV) in red. **(B)** Dose at 2 cm from the PTV (D2cm) not exceeding 50% of the prescribed PTV dose. **(C–E)** Isodose distributions of the normo-fractionated radiotherapy plan **(F)** The accumulative dose of 60Gy and 50Gy was limited to the pretreated area. Light blue (40Gy) and blue (20Gy) correspond to regions treated with normo-fractionated radiotherapy.

After a multidisciplinary discussion and respiratory function assessment (**Table 1**) it was decided to proceed with a new iRT treatment, but outside the context of SBRT. An equivalent dose in 2 Gy fractions (EQD2) with an alpha/beta of 10 (12) was used to reduce the risk of radiotoxicity, prescribing 40 Gy in 20 fractions to achieve volume coverage with VMAT technique, targeting the three sites of oligoprogression. The evaluation of the treatment plan was performed using rigid registration for dose accumulation (**Figure 2**) and biological summation; the data are presented in **Table 2**.

**Table 2:** Cumulative biological dose to organ at risk expressed in EQD2.

Organ at risk*	Dosimetric parameter	Cumulative EQD2	Cumulative EQD2 dose constrains (13)	Percent of cumulative EQD2 dose constrains reached
Left lung	(Dmean; Gy)	6.388	22	29.03%
	V20; %	6.77	40	17%
Right lung	(Dmean; Gy)	8.577	22	38.98%
	V20; %	9.29	40	23%
Lungs	(Dmean; Gy)	7.527	22	34.21%
	V20; 5	6.36	40	16%
Esophagus	(Dmax; Gy)	68.113	75	90.81%
Spinal cord	(Dmax; Gy)	32.412	60	54.02%

EQD2, Equivalent dose in 2 Gy fractions.

*alpha/beta ratio of 2 was used for spinal cord; 2.5 for heart and 3 for esophagus and lung structures.

During treatment, the patient developed Grade 2 pneumonitis (mild cough), according to common terminology criteria for adverse events version 5 (CTCAEv5), and steroid therapy was prescribed.

CR was obtained one month after treatment and it has been maintained until the last follow-up (4 months after treatment, **Figure 1**).

Throughout the treatment period, since 2021, no decline in respiratory function was observed (**Table 1**).

## Discussion

This case report shows how the role of immune-modulant radiotherapy can be harnessed and integrated with IO to achieve prolonged locoregional control of metastatic NSCLC. Here the initial response to pembrolizumab was 12 months, in line with literature reports of PFS (7–9, 14–16).

The median PFS2 (defined as time from randomization to subsequent disease progression after initiation of new anticancer therapy or death from any cause) has been reported in the 5-year analyses of KEYNOTE-024 (15) and KEYNOTE-042 (14) trials as 24.1 months (CI95%: 15 to 31.4 months) and 15 months (CI95%: 11.6 to 19.2 months) respectively. In these trials palliative RT was consented with suspension of IO during RT and continuation afterward (17, 18), but it has not been specified how many patients received RT in this context. KEYNOTE-024 (15) reports that 6.5% of patients received RT as “subsequent therapy” (9.1% in the pembrolizumab group and 6% in the chemotherapy group) without specifying the intent of the treatment, radiation dose, continuation of IO or specific PFS2.

In our report, iSBRT treatment was delivered with a sub-ablative dose (24Gy in 3 fractions; 8Gy/fraction) (1), with the intention not being direct tumor control through RT, but rather to induce an “*in situ* vaccination” (2, 3) that could sustain the effectiveness of IO and achieve tumor control trough pembrolizumab. Recently Dan Duda and colleagues (19) described prospectively that using SBRT with doses <10Gy per fraction increased the proportion of proliferating CD8+ T-cells after the radiotherapy treatment in patients with oligo-metastatic and oligo-progressive pulmonary lesions. In this case, the using of this fractionation may have contributed to observe the abscopal effect that allowed the continuation of pembrolizumab as first-line treatment for up to 45 months (52 cycles).

Considering iSBRT a new anticancer therapy, the PFS2 could be reported as 39 months, longer than the median and confidence interval reported in KEYNOTE-024, and more than twice as long as observed in KEYNOTE-042.

The use of sub-ablative doses has certain advantages: the immunomodulatory effect, already highlighted and exemplified in this case report; the safety of the treatment, especially in patients with pulmonary frailty; and the possibility of re-treatment with an adequate safety profile.

The biological sum of both treatments in the re-irradiated lesion (station 11L) was EQD2: 76 Gy and biologically effective dose (BED): 91.2 Gy (12) with an adequate dose limitation to the organs at risk (OARs) (20), as showed in **Table 2**.

Furthermore, both treatments were delivered without changing respiratory function as presented in **Table 1**.

Finally, it is important to recognize the shift in trend in reported cases of distant immunological effects of RT in NSCLC before and after the use of IO as part of routine clinical practice. Before the introduction of IO, reported cases in NSCLC accounted for only 6% (13), and consequently did not represent a pathology of interest in this area. However, in the IO era, a 116% increase in reported cases has been reported (11, 13), second only to melanoma.

These findings in literature, along with the present case report, suggest that the iRT with sub-ablative doses in oligo-progressive settings may enhance the outcomes already achieved in metastatic NSCLC patients receiving IO.

## Conclusions

We report the induction of a complete response with iRT treatment in combination with pembrolizumab in a patient with metastatic NSCLC. Given its potential impact on survival and prolonged benefit from IO, this strategy warrants further investigation and validation in larger cohorts.

## Data Availability

The original contributions presented in the study are included in the article/supplementary material. Further inquiries can be directed to the corresponding author.
